# The Flagellar Regulator *fliT* Represses *Salmonella* Pathogenicity Island 1 through *flhDC* and *fliZ*

**DOI:** 10.1371/journal.pone.0034220

**Published:** 2012-03-27

**Authors:** Chien-Che Hung, Leanne Haines, Craig Altier

**Affiliations:** Department of Population Medicine and Diagnostic Sciences, Cornell University, Ithaca, New York, United States of America; University of Illinois at Urbana-Champaign, United States of America

## Abstract

*Salmonella* pathogenicity island 1 (SPI1), comprising a type III section system that translocates effector proteins into host cells, is essential for the enteric pathogen *Salmonella* to penetrate the intestinal epithelium and subsequently to cause disease. Using random transposon mutagenesis, we found that a Tn*10* disruption in the flagellar *fliDST* operon induced SPI1 expression when the strain was grown under conditions designed to repress SPI1, by mimicking the environment of the large intestine through the use of the intestinal fatty acid butyrate. Our genetic studies showed that only *fliT* within this operon was required for this effect, and that exogenous over-expression of *fliT* alone significantly reduced the expression of SPI1 genes, including the invasion regulator *hilA* and the *sipBCDA* operon, encoding type III section system effector proteins, and *Salmonella* invasion of cultured epithelial cells. *fliT* has been known to inhibit the flagellar machinery through repression of the flagellar master regulator *flhDC*. We found that the repressive effect of *fliT* on invasion genes was completely abolished in the absence of *flhDC* or *fliZ*, the latter previously shown to induce SPI1, indicating that this regulatory pathway is required for invasion control by *fliT*. Although this *flhDC-fliZ* pathway was necessary for *fliT* to negatively control invasion genes, *fliZ* was not essential for the repressive effect of *fliT* on motility, placing *fliT* high in the regulatory cascade for both invasion and motility.

## Introduction

*Salmonella* is an important bacterial pathogen that is a leading source of food-borne illness, causing diseases ranging from transient enteritis to life-threatening septicemia. To infect its animal hosts, *Salmonella* first must penetrate the intestinal epithelium, a process termed invasion. Most of the genes required for invasion lie within a 40 kb gene cluster at centisome 63 termed *Salmonella* Pathogenicity Island 1 (SPI1), which is used by *Salmonella* to construct a type III secretion apparatus, the needle complex, to deliver secreted effector proteins into the host cell cytoplasm [1], [2], [3], [4], [5], [6]. Once these proteins are translocated into a targeted epithelial cell, they induce cytoskeleton rearrangement and membrane ruffling, resulting in internalization of *Salmonella* by the host cell [7], [8], [9], [10].

SPI1 genes are known to be controlled by several transcriptional regulators encoded within and outside SPI1 through a complex network. Four transcriptional regulators, *hilD*, *hilC*, *hilA* and *invF* are present within SPI1 [4], [11], [12], [13], [14]. Among these, *hilD* is at the top of the regulatory cascade and controls *hilC* as well as a regulator located outside SPI1, *rtsA* [15], [16]. HilD, HilC, and RtsA are able to regulate their own gene expression and can activate expression of *hilD*, *hilC* and *rtsA* independent of each other to constitute a regulatory circuit for the control of the SPI1 central regulator *hilA* [17]. HilA controls the *sic/sip* operon, encoding effector proteins, and the *prg/org* and *inv/spa* operons that encode proteins composing the type III secretion apparatus [13], [14]. HilA also induces the expression of the transcriptional regulator *invF*, encoding a member of the AraC family that activates the expression of genes encoding effector proteins within and outside SPI1 [13], [14]. In addition, *invF* has been shown to be directly regulated by HilD and HilC through a HilA-independent pathway [18]. Several genetic regulators outside SPI1 have also been shown to transcriptionally or post-transcriptionally control invasion gene expression. Regulators affecting SPI1 at the level of transcription include the two-component regulators PhoP/PhoQ, EnvZ/OmpR, PhoB/PhoR and BarA/SirA [1], [19], [20]. In addition, the DNA binding proteins H-NS and Hha have been demonstrated to bind to multiple A-T rich sequences in SPI1, occupying the binding sites of positive regulators, and consequently preventing transcription [21], [22], [23]. Among the post-transcriptional regulators of SPI1, the Csr system, PNPase, Lon protease and HilE have been shown to control invasion genes by affecting protein production or by manipulating the level or activity of HilD [24], [25], [26], [27], [28].

In addition to the mechanisms of control described above, two regulators of the flagellar regulon, *flhDC* and *fliZ*, have been described as inducers of SPI1 [19], [29]. In the *Salmonella* flagellar regulatory cascade, the FlhD_4_C_2_ complex, encoded by *flhDC*, functions as a master regulator that binds to the class 2 flagellar promoters and to its own promoter to induce downstream flagellar gene expression [30], [31]. However, the function of FlhD_4_C_2_ is antagonized by another flagellar protein, FliT, which associates with FlhD_4_C_2_ and neutralizes its activity [32], [33]. *fliZ* has been characterized as a class 2 flagellar gene [34]. Previous studies have shown that mutation of *fliZ* significantly reduces *hilA* expression and *Salmonella* intestinal colonization in mice. In addition, over-expression of *fliZ* increases *hilA* expression only when *hilD* is present, indicating that *fliZ* controls invasion gene expression through *hilD* [29]. Although *fliZ* has been demonstrated to negatively control *hilD*, the mechanism by which this is accomplished remains uncertain [29], [35].

Expression of invasion genes can also be induced using various laboratory conditions that mimic the host intestinal environment, such as low oxygen, high osmolarity, and a near neutral pH [36], [37], [38]. In addition, short-chain fatty acids, produced by the intestinal microbiota through fermentative metabolic pathways, have been shown to play important roles in controlling *Salmonella* invasion [39], [40]. Among these, butyrate, which exists in high concentration in the large intestine where salmonellosis rarely occurs, represses SPI1 gene expression [40], [41].

To identify additional genetic elements involved in *Salmonella* invasion control, here we applied a transposon mutagenesis approach and identified a mutation in the flagellar gene *fliT* that affects the expression of SPI1 genes. As *fliT* was known to be a negative regulator of the flagellar regulon, we used genetic approaches to study the role of *fliT* and associated regulatory elements in the repression of invasion. Here, we demonstrate that *fliT* controls *Salmonella* invasion genes through *flhDC* and the *flhDC*-regulated gene, *fliZ*.

## Results

### Identifying *fliT, a* novel negative regulator of *Salmonella* invasion, using random transposon mutagenesis screening

**Figure 1:**
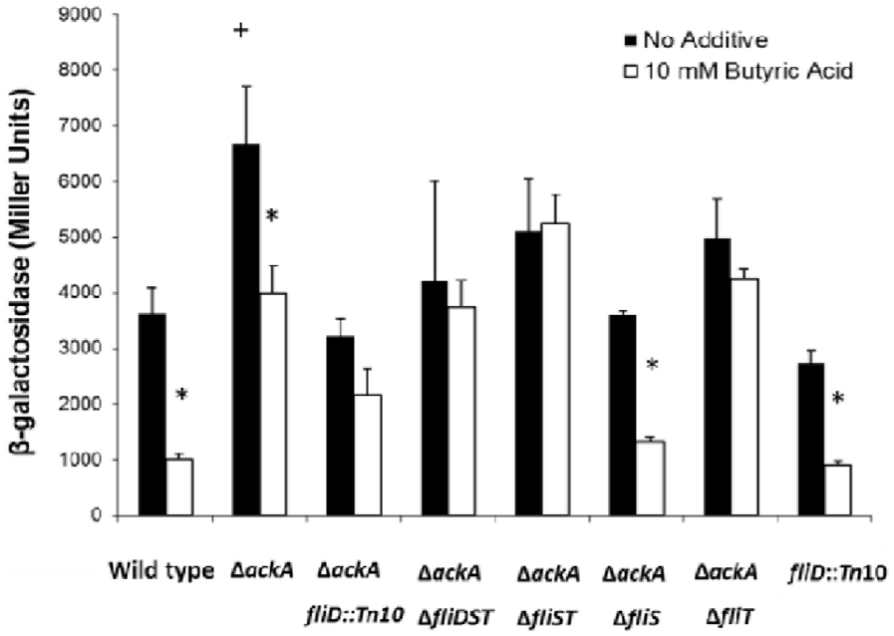
Mutation of *fliT* restores *sipC* invasion gene expression in the *ackA* mutant under SPI1-repressing conditions. Wild type and mutants strains carrying the *sipC*::*lacZY* fusion were grown in LB broth buffered to pH 6.7 with 100 mM MOPS overnight without aeration with no additive (black bars) or with 10 mM butyric acid (white bars). *sipC::lacZY* expression was measured using β-galactosidase assays. The value of individual bars represents means for samples tested in triplicate, and the error bars represent standard deviations. An asterisk (*) indicates a statistically significant difference due to butyrate as compared to the same strain with no additive at p<0.05. A plus (+) indicates a statistically significant difference due to deletion of a gene as compared to the wild type when grown under the same conditions at p<0.05.

*Salmonella* Pathogenicity Island 1 (SPI1) gene expression is controlled by various regulatory elements inside and outside the island, and is also affected by environmental cues [42]. To identify novel regulators that negatively control *Salmonella* invasion, we used random Tn*10* transposon mutagenesis in a strain carrying a *gfp* reporter fusion to the SPI1 gene *sipC*, with the strain grown in the presence of butyrate, a short-chain fatty acid found in abundance within the mammalian intestine. As butyrate has been shown to repress SPI1 genes [40], [41], the bacterial colonies carrying the *sipC::gfp* reporter showed little fluorescence on LB agar containing 10 mM butyric acid. We surmised that transposon insertions in negative regulators of invasion would increase *sipC::gfp* expression, producing fluorescent colonies. The strain used for this screen also carried a deletion of *ackA*, encoding acetyl kinase, as our studies showed that the *ackA* mutation partially restored *sipC* expression in media containing butyric acid (Fig. 1 and data not shown). This strain allowed the screen to be performed without the repeated isolation of *ackA* mutants, and thus provided the possibility of identifying novel regulators of invasion. In total, we screened approximately 40,000 colonies, representing an 8-fold screening of the genome, with 31 fluorescent colonies being found. We next sought to determine the transposon insertion sites in candidate mutants. Previously, it had been reported that Tn*10* insertions near the promoter region of the SPI1 regulator *hilD* could cause increased expression of the downstream regulator *hilA*, which is essential to induce *sipC* [43]. To rule out these and other potential mutations within SPI1, we examined the linkage of Tn*10* insertions to *sipC* using P22 bacteriophage-mediated transductional mapping. The results showed that 22 candidates possessed a Tn*10* insertion linked to *sipC*; these mutants were not further characterized.

For the remaining nine candidate colonies, the Tn*10* insertions were moved by transduction into an *ackA* mutant carrying a MudJ insertion encoding a *lacZY* fusion to the *sipBCDA* operon to quantify the increase in invasion gene expression using β-galactosidase assays. Based upon the increased level of *sipBCDA::lacZY* expression, candidates were categorized into two classes; those with increased expression only when butyrate was present (six mutants), and those with increased expression under both repressing and inducing conditions (three mutants). As individual mutants in each group possessed a similar effect on *sipBCDA* expression, their phenotypes suggested that they might carry Tn*10* disruptions in the same gene or operon. To identify the sites of transposon insertion, we amplified the region flanking the Tn*10* for one candidate from each of the two groups by arbitrary PCR [44]. We found that the mutant affected only under repressing conditions, in the presence of butyrate, carried a Tn*10* insertion in *fliD*, the first gene of the *fliDST* operon. A representative of the second class, showing increased *sipC* expression under both repressing and inducing conditions, carried a Tn*10* insertion in *pnp*. Further, we determined the genetic linkage of Tn*10* in all of the remaining candidates of both groups to *fliD* and *pnp* by transductional mapping, finding that all insertions within a group were 100% linked to these respective genes. These results, taken together, demonstrate that all of the mutations residing outside SPI1 that induced the expression of *sipC* under our tested conditions resulted from disruptions in or near either *fliD* or *pnp*.

### *fliT* is a negative regulator of *Salmonella* invasion

*pnp*, encoding a 3- to 5-phosphorolytic exonuclease, a subunit of RNA degradosome, has been shown to affect SPI1 genes expression by interfering with RNA half-life [28]. However, genes in the *fliDST* operon have not been reported to control invasion by *Salmonella*. For this reason, we focused our study on the role of the *fliDST* operon in the control of SPI1. To quantify the effects of the Tn*10* disruption of *fliD* on invasion, we compared *sipC::lacZY* expression in various mutants grown with or without butyric acid by β–galactosidase assays. In the wild type, *sipC* expression decreased 3.5-fold when the strain was grown in media containing 10 mM butyric acid compared to media with no additive (with all media stably buffered to pH 6.7), and an *ackA* mutant, as expected, demonstrated a lesser, 1.5-fold repression due to the presence of butyric acid (Fig. 1). Importantly, *sipC* expression was unaffected by butyric acid in the *ackA*, *fliD::*Tn*10* double mutant (Fig. 1). As *fliD* is the first gene in the *fliDST* operon, the increase of *sipC* expression caused by the disruption of *fliD* compared to the wild type grown under the same repressive conditions may have resulted from polar effects on any of the downstream genes in the operon. Thus, we next determined which genes played important roles in control of *sipC* expression by testing the effects of mutations of operon genes, singly and in combination. The results showed that *ackA* strains with an additional deletion of *fliDST*, *fliST*, or *fliT* restored *sipC* expression in the presence of butyric acid compared to the same strains without additive (Fig. 1). There remained, however, a significant decrease in *sipC* expression by butyrate in strains with disruptions of *fliD* (data not shown) or *fliS* (Fig. 1), the first two genes of the *fliDST* operon. From these results, we concluded that the last gene of the *fliDST* operon, *fliT*, is required for the negative control of SPI1 gene expression. The increased *sipC* expression caused by deletion of *fliT* was, however, seen only in the *ackA* mutant and with the repression of SPI1 genes provided by butyric acid (Fig. 1). In addition to the repressive effects that required *fliT*, we also found that the loss of some members of the operon in the *ackA* null strain reduced *sipC* expression irrespective of the media conditions employed (Fig. 1; compare white bars to each other). This suggests that components of this operon under some conditions may exhibit a positive effect on invasion gene expression, but in this work we further examined only the genesis of invasion gene repression caused by these genes.

**Figure 2:**
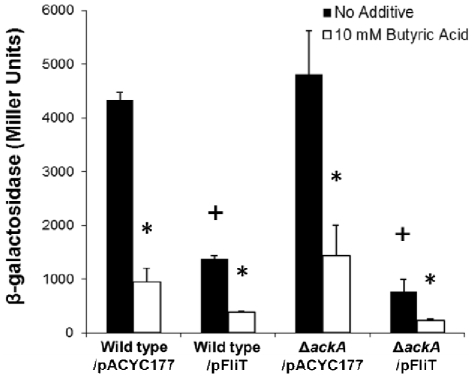
Over-expression of *fliT* negatively controls *sipC* invasion gene expression. The expression of *sipC::lacZY* was measured in strains carrying a low copy-number plasmid expressing *fliT* (pFliT) or its vector control (pACYC177). Bacterial strains were cultured in LB broth buffered to pH 6.7 with 100 mM MOPS overnight without aeration with no additive (black bars) or with 10 mM butyric acid (white bars). *sipC::lacZY* expression was measured using β-galactosidase assays. The value of individual bars represents means for samples tested in triplicate, and the error bars represent standard deviations. An asterisk (*) indicates a statistically significant difference due to butyrate as compared to the same strain with no additive at p<0.05. A plus (+) indicates a statistically significant difference due to pFliT as compared to the isogenic strain carrying the control plasmid pACYC177 at p<0.05.

**Figure 3:**
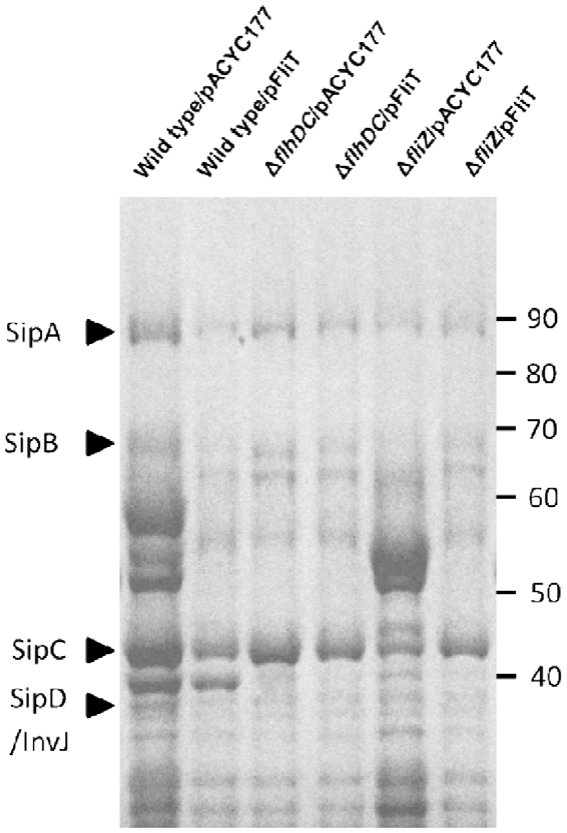
Over-expression of *fliT* decreases SPI1 effector protein production. The wild type, the *flhDC* mutant and the *fliZ* mutant carrying the control plasmid pACYC177 (lanes 1, 3 and 5) or the *fliT* expression plasmid pFliT (lanes 2, 4 and 6) were grown in LB broth with 100 mM MOPS, pH 6.7, and 100 µg/ml ampicillin under low aeration (60 rpm) conditions overnight. Proteins secreted into the culture media were purified as described and separated using 10% SDS-PAGE. The locations of four SPI1 effector proteins, SipA (89 kDa), SipB (67 kDa), SipC (42 kDa) and SipD/InvJ (38 kDa), are shown on the left. Molecular weights (kDa) are shown on the right.

**Figure 4:**
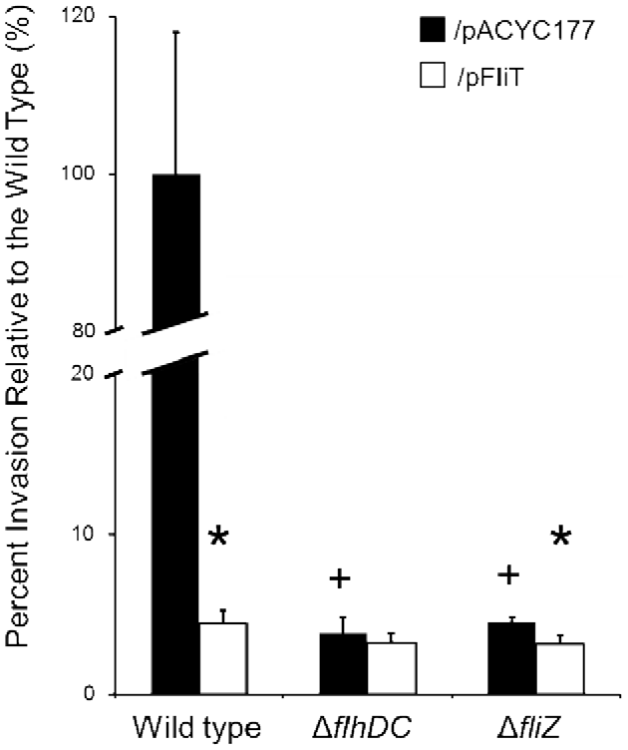
Over-expression of *fliT* negatively controls *Salmonella* invasion. Strains (the wild type, the *flhDC* mutant, and the *fliT* mutant) carrying the *fliT* expression plasmid pFliT (white bars) or control plasmid pACYC177 (black bars) were grown overnight without aeration in LB with 100 mM MOPS, pH 6.7, and 100 µg/ml ampicillin. Invasion of HEp-2 cells by each strain (MOI≈10) was assessed using a gentamicin protection assay. Invasion of all strains is shown relative to the wild type carrying the control plasmid, which was set to 100%. The value of individual bars represents means for samples tested in quadruplicate, and the error bars represent standard deviations. An asterisk (*) indicates a statistically significant difference due to the pFliT plasmid compared to the same strain with the control plasmid at p<0.05. A plus (+) indicates a statistically significant difference due to deletion of a gene as compared to the wild type at p<0.05.

To confirm the negative effect of *fliT* on invasion, we cloned the *fliT* ORF onto a low-copy number plasmid, on which *fliT* was constitutively expressed under the control of an exogenous promoter. Again using a *sipC::lacZY* fusion, we found a significant 3.1-fold decrease in *sipC* expression in the wild type strain with the *fliT* plasmid compared to the isogenic strain carrying the control plasmid, pACYC177 (Fig. 2). This repressive effect of *fliT* on invasion gene expression, however, was limited to conditions of over-expression as a *fliT* mutant carrying a *sipC*::*lacZY* fusion in an otherwise wild type background and grown under SPI1-inducing conditions demonstrated no significant change in *sipC* expression (data not shown), identical to the phenotype of the *fliD::*Tn*10* insertion shown in Figure 1. To verify that the repressive effect of *fliT* on gene expression manifested itself as a significant virulence phenotype, we next characterized changes in the levels of effector proteins of SPI1 produced and secreted by this strain. SPI1 invasion proteins encoded by the *sipBCDA* operon have been shown to be secreted into the culture medium when *Salmonella* is grown in laboratory media [45]. We extracted the secreted proteins from overnight bacterial cultures and examined the SPI1 effector protein profile using SDS-PAGE with Coomassie blue staining (Fig. 3). Four bands had molecular weights equivalent to the invasion proteins SipA (89 kDa), SipB (67 kDa), SipC (43 kDa) and SipD/InvJ (38 kDa) (protein sequences of these bands were determined by mass spectrometry, with the band for SipD overlapping that of another invasion protein, InvJ, due to their similar molecular weights). These bands were significantly diminished in the wild type strain carrying the *fliT* plasmid compared to the strain with the control plasmid (Fig. 3, lanes 1 and 2). In addition, we examined the invasion of the wild type strain carrying the *fliT* plasmid using a gentamicin protection assay with the HEp-2 epithelial cell line. We found that over-expression of *fliT* significantly reduced the ability of *Salmonella* to penetrate these cells, reducing invasion by 23-fold (Fig. 4). Based upon the results of β-galactosidase assay, the secreted protein profile assays, and this invasion assay, we thus demonstrated that *fliT* acts as a repressor of SPI1 gene expression and *Salmonella* invasion when it is over-expressed.

The fact that the loss of *fliT* in the *ackA* deletion mutant could relieve the butyrate–induced repressive effect on the SPI1 gene *sipC* (Fig. 1) led us to speculate that butyrate might function through the induction of *fliT* itself. To test this hypothesis, we used a *fliT*-*lacZ* transcriptional fusion in the wild type and the *ackA* mutant, and examined whether *fliT* expression was increased by butyrate. We found that there was no significant difference in *fliT* expression in either strain background with the addition of butyric acid (data not shown), indicating that *fliT* expression is not affected by butyrate at the transcriptional level. To further investigate whether the negative effects of *fliT* and butyrate on invasion genes were independent, we determined whether the addition of butyrate promoted the repressive effect on SPI1 when *fliT* was over-produced. We over-expressed *fliT* in the wild type and the *ackA* mutant strains carrying the *fliT* plasmid and compared *sipC* expression with or without the addition of butyric acid. As expected, we found that there was a significant further reduction of *sipC* expression by butyrate in these strains, 3.6-fold for the wild type and 3.3-fold for the *ackA* mutant (Fig. 2). These results therefore demonstrate that *fliT* is not involved in the negative control of butyrate on SPI1 gene expression.

### *fliT* negatively controls invasion genes through the flagellar regulators *flhDC* and *fliZ*

**Figure 5:**
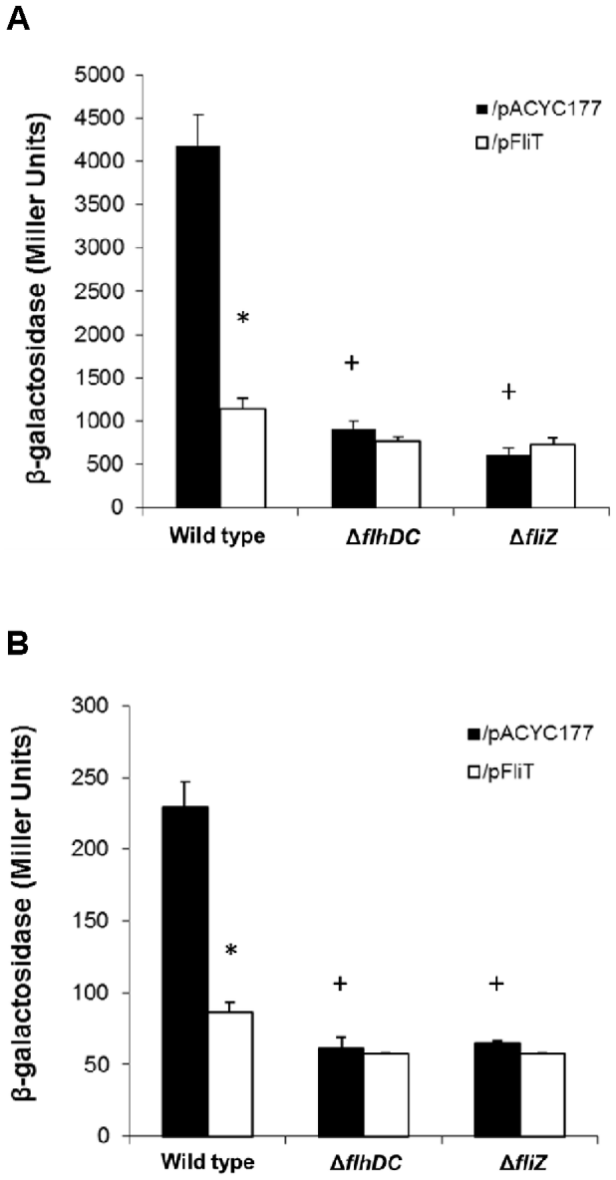
*fliT* affects SPI1 gene expression through the *flhDC-fliZ* pathway. The *fliT* expression plasmid pFliT (white bars) and the control plasmid pACYC177 (black bars) were tested in the wild type, the *flhDC* mutant and the *fliZ* mutant carrying A) the *sipC::lacZY* fusion, and B) the *hilA::lacZY* fusion. Strains were cultured in LB broth with 100 mM MOPS, pH6.7, and 100 µg/ml ampicillin overnight without aeration and *lacZY* expression was measured using β-galactosidase assays. The value of individual bars represents means for samples tested in triplicate, and the error bars represent standard deviations. An asterisk (*) indicates a statistically significant difference due to the *fliT* expression plasmid pFliT as compared to the same strain with the control plasmid at p<0.05. A plus (+) indicates a statistically significant difference due to deletion of a gene as compared to the wild type at p<0.05.

Having shown that over-expression of *fliT* from an exogenous promoter repressed invasion, we further asked how this member of the flagellar regulon exhibited this control. FliT has been shown to function as a chaperone to facilitate export of the flagella capping protein, FliD, in the assembly of flagella [46], [47]. More importantly, FliT has also been demonstrated to negatively control flagellar gene expression by binding to the class 1 flagellar regulator, the FlhD_4_C_2_ complex, and preventing this transcriptional activator from binding to class 2 flagellar promoters, consequently reducing downstream flagellar gene expression [32], [33]. Since FliT can function as a negative regulator of the flagellar regulon, it is possible that the repressive effect of FliT on invasion may result from its negative effects on other flagellar genes that can positively control invasion gene expression. In *Salmonella*, two flagellar genes, *flhDC* and the *flhDC*-controlled downstream regulator *fliZ*, have been shown to positively regulate SPI1 [19], [29]. In addition, *fliZ* has been shown to regulate invasion genes through the control of the SPI1 regulator, HilD [29], [35]. To test whether *fliT* affected *Salmonella* invasion through the negative control of this *flhDC*-*fliZ* pathway, we first examined the abilities of the *flhDC* and *fliZ* mutants, each carrying the *fliT* plasmid or the control plasmid pACYC177, to invade cultured HEp-2 epithelial cells using a gentamicin protection assay (Fig. 4). We found that there was a significant reduction in *Salmonella* invasion, 26-fold for the *flhDC* mutant and 22-fold for the *fliZ* mutant (Fig. 4). These results demonstrate that these two flagellar genes are positive regulators of invasion, and are consistent with results published by other groups [19], [29]. We also found that there was no significant difference in invasion between the *flhDC* mutant carrying the *fliT* plasmid or the control plasmid. However, a 1.4-fold decrease in invasion was observed in the *fliZ* mutant carrying the *fliT* plasmid compared to the same strain carrying the control plasmid (Fig. 4). These results suggested that *flhDC* is required for *fliT* to control invasion, but *fliZ* may be dispensable. As flagella have been shown to affect *Salmonella* invasion [48], mutation of the master flagellar regulator *flhDC* might cause greater effects on flagella production than mutation of *fliZ*, and consequently affect the results of invasion assays. Therefore, to more precisely test whether *fliT* affected invasion genes through the negative control of this *flhDC*-*fliZ* pathway, we next examined *sipC* expression in the wild type, the *flhDC* mutant, and the *fliZ* mutant, each carrying the *fliT* plasmid or the control plasmid. Using the *sipC*::*lacZY* fusion, there was a significant reduction of *sipC* expression, in the *flhDC* mutant (4.6-fold) and the *fliZ* mutant (6.8-fold) (Fig. 5A). Additionally, over-expression of *fliT* did not further reduce *sipC* expression in the *flhDC* or the *fliZ* mutant (Fig. 5A). These results suggest that *fliT* negatively controls *sipC* through this recognized pathway of regulation. To confirm the negative effect of *fliT* on SPI1 genes through *flhDC* and *fliZ*, we further examined the secreted invasion protein profiles using culture conditions identical to those employed for the β-galactosidase assays. The result showed that the secreted invasion effector proteins SipA, SipB, SipC and SipD were significantly diminished in the *flhDC* and the *fliZ* mutants compared to the wild type (Fig. 3, lanes 1, 3 and 5). Additionally, there was no further reduction in these proteins in the *flhDC* or *fliZ* mutant carrying the *fliT* plasmid (Fig. 3, lanes 4 and 6). As we had shown that downstream SPI1 effector proteins were affected by over-expression of *fliT*, in parallel we also determined whether their upstream regulator, *hilA*, was affected. As for the previous β-galactosidase results using *sipC*, *hilA* expression was significantly reduced in the *flhDC* and *fliZ* mutants carrying the control plasmid compared to the wild type with the same plasmid. A 2.8-fold decreased in *hilA* expression was also observed due to the expression of *fliT*, and there was no additional decrease in *hilA* expression in the *flhDC* and *fliZ* mutants carrying the *fliT* plasmid compared to the same strains with the control plasmid (Fig. 5B). Based upon these results, we conclude that *fliT* negatively affects *Salmonella* invasion gene expression through *flhDC* and *fliZ*.

### The *flhDC-fliZ* pathway is required for the repressive effects of *fliT* on invasion gene expression, but not for its effects on flagellar regulation

**Figure 6:**
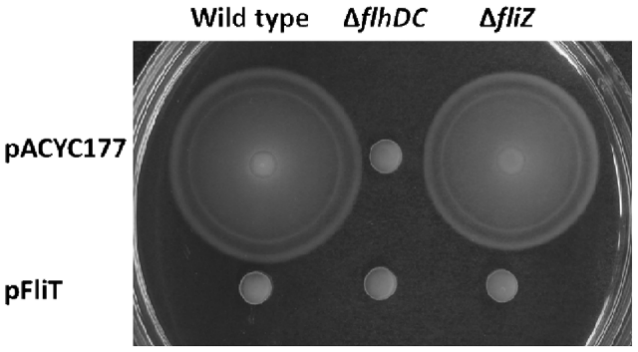
*fliZ* is not required for the negative effects of *fliT* on *Salmonella* motility. The wild type, the *flhDC* mutant and the *fliZ* mutant carrying the control plasmid pACYC177 or the *fliT* expression plasmid pFliT were grown in LB broth with aeration overnight. Cultures were dotted onto LB swimming agar plates (0.35% agar) with 100 µg/ml ampicillin and incubated at 37°C for 7 hours in a humidified incubator.

Our results demonstrate that *fliT* acts as a negative regulator of invasion, and previous studies have shown that *fliT* affects flagellar control in *Salmonella* [32], [33], [49]. Additionally, our data suggest that the repressive effect of *fliT* on invasion genes is accomplished through the *flhDC*-*fliZ* pathway. Since *flhDC* and *fliZ* have been implicated as regulators in the flagellar regulon [50], we further asked whether this *flhDC*-*fliZ* route is used by *fliT* in its control of flagella. To test this, we used the wild type, the *flhDC* mutant, and the *fliZ* mutant carrying either the control plasmid or the *fliT* plasmid, and examined their swimming ability on 0.35% LB agar plates (Fig. 6). We found that in the wild type strain over-expression of *fliT* completely eliminated *Salmonella* motility. The same phenotype was also observed in the *flhDC* mutant whether it carried the control plasmid, pACYC177, or the *fliT* plasmid. As previously described, *fliT* is able to negatively control flagellar gene expression by post-translational regulation of FlhD_4_C_2_ activity. Our results thus suggest that *fliT* controls *Salmonella* motility through *flhDC*, and are consistent with other studies. However, unlike *flhDC*, the *fliZ* mutant showed only a slight reduction in swimming ability, and over-expression of *fliT* in this strain fully inhibited its motility, suggesting that the repression of motility by *fliT* was not mediated through *fliZ*. Therefore, our results, taken together, suggested that the *flhDC-fliZ* pathway is specific for repression of *Salmonella* invasion gene expression by *fliT*, but this pathway is not required for *fliT* repression of the flagellar regulon.

## Discussion

For serovars of *Salmonella*, the genes of SPI1 are key elements that dictate the ability of the pathogen to penetrate the intestinal epithelium and cause further systemic infection. The control of SPI1 gene expression has been shown to be evoked by complex interrelated regulatory networks. In this work, using a random transposon mutagenesis strategy, we discovered that the flagellar regulator *fliT*, encoded within the *fliDST* operon, can negatively control SPI1 gene expression (Fig. 1). In addition, we showed that *fliT* over-expression reduced invasion gene expression (Figs. 2, 3, and 5) and *Salmonella* invasion (Fig. 4), and that this repressive effect on SPI1 functioned through the negative control of the *flhDC-fliZ* pathway (Figs. 3 and 5). *fliZ* has been shown to positively control invasion genes by regulating the SPI1 regulator HilD, which exists high in the regulatory hierarchy of this pathogenicity island [16], [29]. It has been suggested that FliZ post-transcriptionally controls HilD through an unidentified mechanism, rather than affecting *hilD* expression at the level of transcription [29]. Kage and coworkers showed that HilD protein level, when expressed from a constitutive promoter, was significantly decreased by the deletion of *fliZ*. However, the half-life of HilD was not changed when *fliZ* was missing. Their studies thus suggest that *fliZ* controls HilD at the translational level [35]. In contrast, Chubiz and colleagues showed that HilD, when constitutively expressed from a single-copy chromosomal tetracycline-inducible promoter, was only slightly reduced in the *fliZ* null strain compared to that in the wild type. Additionally, they measured the stability of HilD in the *fliZ* mutant and in the wild type and showed that the stability of HilD was not significantly altered in this mutant. Therefore, they suggested that the mechanism by which *fliZ* regulates HilD is by post-translationally affecting HilD activity [29]. As we have shown that *fliT* negatively controls invasion through *fliZ*, we suggest that *fliT* negatively controls *Salmonella* invasion by changing the amount or activity of HilD, and subsequently affects expression of downstream invasion genes.

FliT has been shown to possess two functions in *Salmonella*, acting both as a regulator and a chaperone [46], [49], [51]. In its chaperone function, FliT directly interacts with several flagellar proteins, including FliD, FliI and FliJ, preventing their pre-maturation and aggregation within the bacterial cytoplasm and thus facilitating flagellar assembly [46], [47]. As a regulator of flagellar expression, FliT binds to FlhC as part of the FlhD_4_C_2_ complex and inhibits FlhD_4_C_2_ from binding to its target promoters, consequently repressing downstream flagellar gene expression [32]. In our work, we showed that *fliT*, when deleted, was the only gene of the *fliDST* operon able to restore invasion gene expression under our test conditions. We would expect that if FliT had affected invasion genes through its role as a chaperone, by interacting with FliD, the deletion of *fliD* would similarly restore *sipC* expression, as did the *fliT* mutant (Fig. 1). However, restoration of *sipC* expression was not observed in the *fliD* mutant under the conditions used (data not shown), suggesting that FliT does not affect SPI1 gene expression through its function as a chaperone. Instead, our results indicate that *fliT* acts on invasion genes in its role as a regulator, as we have demonstrated that the *flhDC-fliZ* pathway with which FliT is known to interact is required for its negative control of invasion genes. In addition to the repressive effects that we identified for *fliT*, we also found that the loss of some members of the *fliDST* operon caused a reduction in *sipC* expression when tested in the *ackA* null mutant, a phenotype that was independent of the media conditions used (Fig. 1). This may suggest that different components of this operon can have opposing actions. Specifically, it is possible that *fliT* functions as a repressor, but that other products of the operon, alone or in combination with *fliT*, might act as inducers. Work in this area will require further efforts.

In agreement with previous studies [32], [33], our work suggests that *flhDC* is also required for *fliT* to control *Salmonella* motility (Fig. 6). However, we found that *fliZ* was required for the effects of *fliT* only on SPI1 gene expression, and not on motility, as only a slight reduction in swimming was observed in the *fliZ* mutant (Fig. 6). These results, taken together, thus demonstrate that *fliT* is able to coordinately regulate invasion and flagellar gene expression through the single flagellar master regulator *flhDC*, but that the control of these two regulons diverges at subsequent steps in their regulatory cascades.

In *Salmonella*, flagella and invasion have been shown to be coordinately regulated by regulators in addition to *fliT* through the *flhDC-fliZ* pathway. ClpXP is an ATP-dependent protease and has been demonstrated to repress both flagellar and SPI1 gene expression [35]. ClpXP negatively controls the flagellar regulon by facilitating the degradation of the master flagellar regulators FlhD and FlhC and subsequently repressing downstream flagellar genes [52]. Kage and coworkers have demonstrated that the repressive effect of ClpXP on the *flhDC*-*fliZ* cascade is required for this protease to negatively control invasion genes [35]. TviA is another regulator that negatively co-regulates invasion and flagellar genes through this pathway. TviA is a regulator within *Salmonella* Pathogenicity Island 7(SPI7) unique to *Salmonella* serovar Typhi that does not exist in *S*. Typhimurium. This regulator has been shown to respond to stimulation by low osmolarity and also negatively controls both flagellar and invasion gene expression. TviA affects flagellar genes by repressing the transcription of *flhDC*. This inhibitory effect on *flhDC* has been suggested to consequently cause the reduction of invasion gene expression through the *flhDC–fliZ* pathway [53]. The above two regulators and FliT have thus been demonstrated to either transcriptionally or post-translationally affect the flagellar master regulator *flhDC*, while previous studies and the results we present here demonstrate that the *flhDC*-*fliZ* pathway is essential for these regulators to control invasion genes. Based on this evidence, we suggest that the *flhDC–fliZ* pathway is an important common route used by *Salmonella* to allow the flagellar regulon to coordinately control invasion gene expression.

As previously described, FliT has been shown to negatively control FlhD_4_C_2_ activity by its interaction with FlhC and subsequently to inhibit the binding of FlhD_4_C_2_ to target DNA [32]. Interestingly, Aldridge and coworkers showed that FliT was able to interact with FlhD_4_C_2_ that has not bound to its target DNA leading to the dissociation of the FlhD_4_C_2_ complex *in vitro* [33]. However, when FlhD_4_C_2_ was pre-associated with its target DNA, this protein-DNA complex was insensitive to FliT [33]. Their studies suggest a means by which *Salmonella* can efficiently control the flagellar regulon in response to rapidly changing environments. When FliT is produced, it binds existing FlhD_4_C_2_, dissociating the FlhD_4_C_2_ complex and resulting in a quick down-regulation of flagellar gene expression. When the level of FliT is low, however, the FlhD_4_C_2_ complex associates with its target DNA and thus efficiently activates the flagellar regulon. A recent study has also shown that flagella and invasion are coordinately regulated in response to growth phase. Both are highly expressed in the early stages of growth in laboratory medium [54]. However, these two regulons were both repressed in late stationary phase, and alternatively fimbrial genes were highly expressed [54]. This phenomenon may be relevant to the control of *Salmonella* gene expression within the intestine of an animal host. Infecting bacteria first utilize flagella and express invasion genes to reach and invade the intestinal epithelium. For those unable to penetrate the epithelium, expression of fimbrial genes would allow bacteria to better colonize within the intestine [54]. Thus, although the environmental and genetic cues of the intestinal tract that elicit control of flagella and invasion, including that mediated by *fliT*, are not well known, the coordinated regulation of these two important functions is clearly essential to productive infection and disease.

## Materials and Methods

### Construction of mutant strains

*Salmonella enterica* serovar Typhimurium strain ATCC 14028S and isogenic mutants were used throughout this study, and are shown in Table 1. Gene deletions were made using the previously reported one-step inactivation method [55]. In brief, PCR reactions were performed to amplify the fragments containing the FRT sequences flanking the antibiotic resistance markers from plasmids pKD3 or pKD4 using primers carrying 40 bases of homologous sequence flanking the coding region of the target gene. The resulting PCR product was purified and transformed into a *Salmonella* strain carrying the plasmid pKD46, which expresses the Red λ recombinase, allowing allelic exchange. The resulting deletion mutants were cultured at 42°C to remove the temperature-sensitive pKD46 plasmid, and the loss of the target gene was determined by PCR. The chromosomal *sipC::gfp* translational fusion was created using the above one-step gene exchange method. A promoterless *gfp* linked to a chloramphenicol resistance marker was amplified from the plasmid pZEP07 [56] with primers TGAGACGTTGATCGGCACGTAAGAGGTTCCAACTTTCACCTGTAGGCTGGAGCTGCTTCG and TTAAATCACACCCATGATGGCGTATAGATGACCTTTCAGACATATGAATATCCTCCTTAG, which encode DNA homologous to the regions immediately adjacent to the *sipC* open reading frame. The resulting PCR product was purified and treated with *Dpn*I to remove the pZEP07 template and transformed into the *Salmonella* strain carrying pKD46 with selection on 25 µg/ml chloramphenicol to allow recombination of *gfp*, creating a translational fusion to *sipC* with an adjacent chloramphenicol cassette. To create the *fliT* expression plasmid (pFliT), a PCR product was produced that included a synthetic ribosome binding site, based upon that of *lacZ*, and the *fliT* ORF with an additional 175 bp 3′ of the *fliT* sequence to include the predicted transcriptional termination site. This product was amplified using primers CCCATCGATCAATTTCACACAGGAAACAGCTATGACCTCAACCGTGGAGTTTATCAAC and TCCCCCGGGGATATCATTCAGCCCATCAGCACG. The PCR product was then cloned into the unique *Cla*I and *Sma*I sites of pACYC177 to place *fliT* under the control of the kanamycin resistance gene (*npt*) promoter on this vector.

**Table 1:** Strains and plasmids used in this study.

Strain or Plasmid	Genotype	Source or reference
Strains		
*Salmonella enterica* serovar Typhimurium 14028S	wild type	American Type Culture Collection
CA412	*sipC::lacZY*	[36]
CA2312	Δ*ackA sipBCDA::*MudJ	This study
CA2311	Δ*ackA sipC::gfp*	This study
CA1274	Δ*ackA sipC::lacZY*	This study
CA2064	Δ*ackA fliD::*Tn*10 sipC::lacZY*	This study
CA2123	Δ*ackA fliDST::kan sipC::lacZY*	This study
CA2124	Δ*ackA fliST::kan sipC::lacZY*	This study
CA2125	Δ*ackA fliS::kan sipC::lacZY*	This study
CA2126	Δ*ackA fliT::kan sipC::lacZY*	This study
CA2047	*fliD::*Tn*10 sipC::lacZY*	This study
CA2060	*flhDC::cam*	This study
CA1854	*fliZ::kan*	This study
CA2121	*flhDC::cam sipC::lacZY*	This study
CA2122	*fliZ::cam sipC::lacZY*	This study
Plasmids		
pNK2883	Plasmid carrying IPTG-inducible Tn*10* transposon	[57]
pMS421	Plasmid carrying *lacI^q^*	[58]
pZEP07	Plasmid carrying *gfp*	[56]
pACYC177	Cloning vector	[61]
pFliT (pCA173)	*fliT* ORF on pACYC177	This study

### Tn*10* random transposon mutagenesis screening

To create a random transposon Tn*10* library, a wild type strain carrying the IPTG-inducible Tn*10* plasmid pNK2883 [57] and an additional plasmid pMS421 [58] that expresses *lacI^q^* was used. The strain was grown overnight in LB broth with 100 µg/ml of ampicillin, 100 µg/ml of spectinomycin, and 20 µg/ml streptomycin at 37°C with shaking, and sub-cultured in the same medium to mid-log phase. To induce transposon insertion, IPTG was added at a final concentration of 0.1 mM to the mid-log culture, which was grown for another 16 hours. The resulting random Tn*10* insertion library was moved into the *ackA* mutant strain carrying the chromosomal *sipC*::*gfp* translational fusion by P22 phage transduction [59]. Transductants were plated on LB agar with 25 µg/ml tetracycline, 100 mM 3-(N-morpholino)propanesulfonic acid (MOPS) pH 6.7, 10 mM ethylene glycol tetraacetic acid (EGTA), and 10 mM butyric acid and incubated at 37°C overnight. The green florescence of individual colonies was determined using an OV100 Observation Intravital System (Olympus Corp., Tokyo, Japan).

### Determining the DNA sequence flanking the Tn*10* element

The sequences flanking the Tn*10* insertions were identified using a method previously reported [44]. In brief, Tn*10* insertion strains and a control strain (the isogenic strain without a Tn*10* insertion) were grown overnight in LB broth with aeration. The overnight culture was diluted 100-fold with nuclease-free water, and bacteria were frozen and thawed three times to expose the genomic DNA. Five µl of the sample was used as a template to perform an initial PCR using primer AATTGCTGCTTATAACAGGCACTG in combination with arbitrary primers GGCCAGCGAGCTAACGAGACNNNNGTTGC, GGCCAGCGAGCTAACGAGACNNNNGATAT, and GGCCAGCGAGCTAACGAGACNNNNAGTAC with a cycle of 3 min at 95°C followed by 30 cycles of 30 sec denaturation at 95°C, 30 sec annealing at 38°C, 90 sec extension at 72°C and an additional cycle of 3 min final extension at 72°C. Five µl of this PCR reaction was next used as template to perform a second PCR using the primer set GGCCAGCGAGCTAACGAGAC and ACCTTTGGTCACCAAAGCTTT, beginning with a cycle of 3 min denaturation at 95°C followed by 30 cycles of 15 sec denaturation at 95°C, 30 sec annealing at 56°C, 90 sec extension at 72°C and a final cycle of 3 min extension at 72°C. PCR products were separated by electrophoresis on a 2% agarose gel. The DNA fragments produced from the Tn*10* insertion mutants but not from the control strain were harvested from the gel. Purified DNA fragments were sequenced using the primer ACCTTTGGTCACCAAAGCTTT.

### Bacteriophage-mediated transductional mapping

The bacteriophage P22 was used to map the location of genes by transduction [59]. The donor strain carrying the antibiotic marker inserted in the bacterial chromosome was grown in LB overnight at 37°C with aeration. Four hundred µl of culture was added to 2 ml of P22 phage broth containing 5×10^6^ pfu/ml of the phage. The mixture was grown overnight at 37°C with aeration, and 6 drops of chloroform was then added to make the phage lysate. The recipient strain, harboring a different antibiotic marker in the chromosome, was grown in LB to mid-log phase, and 10 µl of phage lysate prepared from the donor strain was added to 500 µl of mid-log culture and incubated for 30 minutes at 37°C. Then, 500 µl of 20 mM EGTA was added to the above mixture and incubated at 37°C for 1 hour. Transductants were selected on LB agar with 10 mM EGTA and the antibiotic to which the donor strain was resistant. Fifty resulting colonies were patched onto LB agar with or without the antibiotic to which the recipient strain was resistant to assess the genetic linkage between the two markers.

### β-galactosidase assays

Triplicate cultures of tested bacterial strains were grown standing overnight at 37°C in LB broth buffered to pH 6.7 with 100 mM MOPS and with 10 mM butyric acid and 100 µg/ml ampicillin if needed. β-galactosidase activity was measured as described previously [60].

### Secreted protein isolation and analysis

Strains were grown in LB with100 mM MOPS, pH 6.7, and 100 µg/ml ampicillin at 37°C with shaking at 60 rpm for 16 hours. Proteins secreted into the culture supernatant were prepared and analyzed as previously described [20].

### HEp-2 cell invasion assays

HEp-2 cells were grown in 24 well plates to confluence (approximately 5×10^5^ cells) in RPMI-1640 with 10% fetal bovine serum. Bacteria were grown overnight as static cultures in LB with 100 mM MOPS, pH 6.7, and 100 µg/ml ampicillin at 37°C. Approximately 5×10^6^ bacteria were added to each well. Plates were centrifuged for 10 min at 800× *g* and incubated for 1 hour at 37°C. Medium was discarded, and the cells were washed three times with 0.5 ml PBS. One ml of cell culture media supplemented with 20 µg/ml gentamicin was added to each well, and the cells were incubated for 1 hour at 37°C to kill the extracellular bacteria. Medium was removed, and the cells were washed three times with 0.5 ml PBS. Then, 200 µl of 1% Triton X-100 in PBS was added to each well for 5 minutes to lyse the cells, and 800 µl of PBS was added to individual wells to produce a final volume to 1 ml. The bacterial titers of the lysate were determined by colony counts. Each bacterial culture was tested in quadruplicate wells.

### Bacterial swimming activity

Strains were grown overnight in LB with 100 µg/ml ampicillin at 37°C with shaking at 200 rpm. Ten µl of overnight culture of each strain was dotted onto the LB swimming agar plates (containing 0.35% agar) with100 µg/ml ampicillin, and incubated at 37°C for 7 hours in a humidified incubator.

### Statistical analysis

Results from β-galactosidase assays and invasion assays were analyzed using a one-way analysis of variance to determine if the mean of at least one strain or condition differed from any of the others. The Tukey-Kramer HSD multiple comparison test was then used to determine which means were statistically different. A p-value<0.05 was considered significant. Statistical analysis was performed using Jmp 9.0 software (SAS, Cary, NC).
